# Supplementary material for: LC-MS Analysis of Serum for the Metabolomic Investigation of the Effects of Pulchinenoside b4 Administration in Monosodium Urate Crystal-Induced Gouty Arthritis Rat Model
Source: Molecules. 2019 Aug 30;24(17):3161. doi: 10.3390/molecules24173161 (PMC6749452; doi:10.3390/molecules24173161)
Supplement: Supplementary file 1 [file molecules-24-03161-s001.pdf]

Supplementary Material:

Table S1. Detailed structure elucidation data (NMR) of P-b4.

**Description(^{1}H-NMR,600 MHz, C5D5N)**
White amorphous powder,^{1}H-NMR (600 MHz, C5D5N) δ: 1.72 (3H, s, H-30), 1.03 (3H, s, H-27), 0.977 (3H, s, H-26), 0.91 (3H, s, H-25), 0.69 (3H, s, H-24), 1.25 (3H, d, J=6.0 Hz, 6-H3 of rha), 1.27 (3H, d, J=6.6 Hz, 6′-H3 of rha), 4,57(1H, d, J=4.8Hz, H-1 of ara), 4.75 (1H, brs, 1′-H of rha), 5.17 (1H, brs, 1-H of rha), 5.48 (1H, d, J=8.4Hz, 1′-H of glc), 4.40 (1H, d, J=7.8 Hz, 1″-H of glc).

**Data(^{13}C-NMR,150 MHz, C5D5N)**			
**Aglycone**		**Glycosyl**	
**Carbon**	**Chemical Shift**	**Carbon**	**Chemical Shift**
**1**	39.33	C3-O-	
**2**	26.48	**Rha-1**	101.73
**3**	81.16	**2**	72.43
**4**	42.84	**3**	72.61
**5**	47.84	**4**	74.19
**6**	18.19	**5**	69.45
**7**	34.31	**6**	18.64
**8**	41.20	**Ara-1**	104.43
**9**	51.01	**2**	75.85
**10**	36.95	**3**	74.83
**11**	21.21	**4**	69.49
**12**	26.08	**5**	65.77
**13**	38.38	C28-O-	
**14**	43.68	Glc-1′	95.33
**15**	30.88	2′	74.15
**16**	32.32	3′	78.20
**17**	57.03	4′	70.88
**18**	47.92	5′	78.07
**19**	49.83	6′	61.35
**20**	150.87	Glc-1″	105.21
**21**	30.26	2′	75.38
**22**	37.07	3′	76.50
**23**	63.95	4′	78.76
**24**	13.87	5′	77.24
**25**	17.04	6′	69.78
**26**	16.47	Rha-1′	102.77
**27**	14.95	2′	72.66
**28**	175.01	3′	72.84
**29**	110.19	4′	74.05
**30**	19.45	5′	70.38
		6′	18.62

Table S2. The R^2 and Q^2 of 12 OPLS-DA models.

**Project**	**R^2X**	**R^2Y**	**Q^2**
POS-C9-D9	0.386	0.999	0.951
POS-C9-M9	0.38	0.996	0.961
POS-C24-D24	0.386	0.995	0.956
POS-C24-M24	0.409	0.989	0.948
POS-D9-M9	0.268	0.984	0.854
POS-D24-M24	0.418	0.988	0.77
NEG-C9-D9	0.536	0.996	0.962
NEG-C9-M9	0.415	0.983	0.939
NEG-C24-D24	0.411	0.999	0.939
NEG-C24-M24	0.358	0.981	0.884
NEG-D9-M9	0.256	0.963	0.757
NEG-D24-M24	0.680	1.000	0.542

Table S3. The Metabolic pathway analysis results (using KEGG pathway database).

Pathway Name	p	-log(p)	Holm p	FDR	Impact
Arachidonic acid metabolism	7.5309E-5	9.4939	0.0061	0.0050608	0.55495
Sphingolipid metabolism	1.2496E-4	8.9875	0.0099966	0.0050608	0.2782
Glycerophospholipid metabolism	0.0068031	4.9904	0.53744	0.18027	0.11203
Valine, leucine and isoleucine biosynthesis	0.0089022	4.7215	0.69437	0.18027	0.66666
Aminoacyl-tRNA biosynthesis	0.058411	2.8402	1.0	0.94626	0.0
Valine, leucine and isoleucine degradation	0.0916	2.3903	1.0	1.0	0.0
Pantothenate and CoA biosynthesis	0.18593	1.6824	1.0	1.0	0.0
Folate biosynthesis	0.19708	1.6241	1.0	1.0	0.0
Biosynthesis of unsaturated fatty acids	0.44105	0.81861	1.0	1.0	0.0
Arginine and proline metabolism	0.45656	0.78404	1.0	1.0	0.08228

Table S4. The verification information of identified metabolites.

NO.	Metabolites	TR/min	m/z	M.F	Adduct Type	Matched MS/MS Pyrolysis Characteristic Fragment	Delta (ppm)	HMDB
**1**	L-valine	9.94	118.0848	$C_5H_{11}NO_2$	M+H	118.0868、100.0762、72.0813、43.0547	10	HMDB0000883
2	2-Methylglutaric acid	9.62	129.0543	$C_6H_{10}O_4$	M+H-H2O	147.0647、131.0344、129.0551、111.0446、101.0602、73.0289	7	HMDB0000422
3	L-Isoleucine	5.29	132.1013	$C_6H_{13}NO_2$	M+H	132.1013、114.3001、86.2330、69.2003	5	HMDB0000172
4	L-Arginine	11.90	175.1186	$C_6H_{14}N_4O_2$	M+H	175.0592、158.7482、140.1545、98.0538、72.0421、59.9551	2	HMDB00517
5	Phosphorylcholine	12.55	184.0731	$C_5H_{15}NO_4P$	M+H	184.0731、175.0233、154.2013、135.0221、73.0014	4	HMDB01565
6	8-Hydroxy-7-methylguanine	11.98	223.0935	$C_6H_7N_5O_2$	M+ACN+H	182.0677、166.0364、155.0569、139.0619、111.0194、86.0354	1	HMDB06037
7	4a-Carbinolaminetetrahydrobiopterin	9.62	281.1355	$C_9H_{13}N_5O_3$	M+ACN+H	240.1096、222.0990、204.0885、166.0728、139.0619、89.0602	1	HMDB02215
8	Oleamide	12.44	282.2798	$C_{18}H_{35}NO$	M+H	282.2796、265.2531、247.2425、181.1956、139.1486、85.1017、57.0704	2	HMDB0002117
9	Sphinganine	12.78	284.2956	$C_{18}H_{39}NO_2$	M+H-H2O	254.7790、95.2531、82.7310、69.5770、54.7791	1	HMDB0000269
10	Glycerophosphorylcholine	22.46	299.1370	$C_8H_{20}NO_6P$	M+ACN+H	258.1101、240.0995、222.0889、155.0103、84.0807、75.0440、57.0334	1	HMDB0000086
11	Phytosphingosine	9.73	318.2972	$C_{18}H_{39}NO_3$	M+H	318.3008、301、2742、282.2796、197.2262、71.0860、43.0183	10	HMDB0004610
12	Geranylgeranyl-cysteine	11.04	408.2590	$C_{23}H_{37}NO_3S$	M+H	408.2566、390.2461、287.2369、245.2263、122.0270、104.0164	6	HMDB0011678
13	Beta-tocopherol	20.28	458.3973	$C_{28}H_{48}O_2$	M+ACN+H	417.3727、399.3621、197.2263、151.0753、	4	HMDB0006335
14	LysoPC(18:2(9Z,12Z))	11.43	502.3289	$C_{26}H_{50}NO_7P$	M+H-H2O	520.3407、502.3303、443、2565、337.2750、184.0735、104.1070	2	HMDB0010386

15	LysoPC(18:1(9Z))	16.79	506.3585	$C_{26}H_{52}NO_6P$	M+H	506.3605、403.2607、323.2945、249.2576、180.0420、88.1120	4	HMDB0010408
16	LysoPC(0:0/18:0)	16.81	524.3676	$C_{26}H_{46}NO_7P$	M+H	524.3710、506.3605、240.0995、184.0733、88.1120、84.0807	7	HMDB0011128
17	LysoPC(20:3(5Z,8Z,11Z))	16.79	528.3437	$C_{28}H_{50}NO_7P$	M+H-H2O	546.3554、363.2893、289.2525、247.2420、240.0995、88.1120	3	HMDB0010393
18	Postin(Lys-Pro-Pro-Arg)	12.42	529.3459	$C_{22}H_{40}N_8O_5$	M+CH3OH+H	480.2928、286.1873、167.1178、101.1073、98.0600、70.0651	0	HMDB0005772
19	LysoPC(22:6(4Z,7Z,10Z,13Z,16Z,19Z)	11.98	550.3278	$C_{30}H_{52}NO_7P$	M+H-H2O	570.3554、387.2893、313.2525、271.2420、240.0995、88.1120	4	HMDB0010404
20	LysoPC(22:5(4Z,7Z,10Z,13Z,16Z)	11.34	552.3448	$C_{30}H_{50}NO_7P$	M+H-H2O	552.3554、387.2891、285.2536、271.2422、240.0975、88.1121	1	HMDB0010402
21	25-Hydroxyvitamin- D2-25-glucuronide	11.98	571.3603	$C_{34}H_{52}O_8$	M+H-H2O	571.3634、413.3419、395.3313、311.2374、277.2531、123.0809	6	HMDB0010342
22	Mesobilirubinogen	11.98	593.3305	$C_{33}H_{44}N_4O_6$	M+H	593.3336、575.3227、547.3278、529.3173、487.3067、124.0756	5	HMDB0001898
23	Kinetensin 4-7	4.82	604.3261	$C_{26}H_{37}N_9O_6$	M+CH3OH+H	555.2674、513.2456、266.1723、207.1240、129.1134、60.0556	10	HMDB0012986
24	Leukotriene C4	4.82	658.3340	$C_{30}H_{47}N_3O_9S$	M+CH3OH+H	608.3005、580.3056、523.2841、495.2528、392.2259、74.0242	4	HMDB0001198
25	Kinetensin 4-8	4.89	719.3584	$C_{35}H_{46}N_{10}O_7$	M+H	718.3551、702.3358、363.2251、266.1723、129.1134、112.0869	6	HMDB0012987
26	PS(14:0/14:1(9Z)	20.85	719.4602	$C_{34}H_{64}NO_{10}P$	M+ACN+H	660.4240、493.4256、211.2061、88.0398、71.0133、42.0343	1	HMDB0012331
27	Sphingosine	5.61	298.1358	$C_{18}H_{37}NO_2$	M-H	298.2746、280.2640、250.2534、60.0449、42.0343	4	HMDB00252
28	3-dehydrosphinganine	18.69	298.3111	$C_{18}H_{37}NO_2$	M-H	298.3106、280.2540、266.2483、250.2534、58.0292、	3	HMDB0001480
29	Arachidonic acid	19.99	303.957	$C_{20}H_{32}O_2$	M-H	303.2342、259.2430、205.1061、59.0169	2	HMDB0001043

30	Leukotriene A4	13.40	317.2079	$C_{20}H_{30}O_3$	M-H	317.2146、299.2048、255.2124、59.0216、	1	HMDB01337
31	12-Keto-tetrahydro-leukotriene B4	12.93	319.2266	$C_{20}H_{34}O_4$	M-H2O-H	337.2384、293.2486、275.2380、221.1183、155.1443、59.0138	2	HMDB02995
32	5(S)-Hydroperoxyeicosatetraenoic acid	10.93	335.2189	$C_{20}H_{32}O_4$	M-H	335.2130、317.2334、289.2373	2	HMDB0001193

Table S5. CV-ANOVA of 12 OPLS-DA score plots.

POS-C9-M9	SS	DF	MS	F	p	SD	NEG-C9-M9	SS	DF	MS	F	p	SD
Total corr.	19	19	1			1	Total corr.	19	19	1			1
Regression	18.27	4	4.57	93.50	2.04E-10	2.14	Regression	17.77	4	4.44	54.34	9.52E-09	2.11
Residual	0.73	15	0.049			0.221	Residual	1.23	15	0.082			0.286
**POS-C9-P9**	**SS**	**DF**	**MS**	**F**	**p**	**SD**	**NEG-C9-P9**	**SS**	**DF**	**MS**	**F**	**p**	**SD**
Total corr.	19	19	1			1	Total corr.	19	19	1			1
Regression	18.07	6	3.01	42.28	8.66E-08	1.74	Regression	18.28	6	3.05	55.17	1.69E-08	1.75
Residual	0.93	13	0.071			0.267	Residual	0.72	13	0.055			0.235
**POS-P9-M9**	**SS**	**DF**	**MS**	**F**	**p**	**SD**	**NEG-P9-M9**	**SS**	**DF**	**MS**	**F**	**p**	**SD**
Total corr.	19	19	1			1	Total corr.	19	19	1			1
Regression	16.22	4	4.06	21.92	4.01E-06	2.01	Regression	14.39	4	3.60	11.71	1.63E-05	1.90
Residual	2.78	15	0.185			0.430	Residual	4.61	15	0.307			0.554
**POS-C24-M24**	**SS**	**DF**	**MS**	**F**	**p**	**SD**	**NEG-C24-M24**	**SS**	**DF**	**MS**	**F**	**p**	**SD**
Total corr.	19	19	1			1	Total corr.	19	19	1			1
Regression	18.01	4	4.50	67.90	1.99E-09	2.12	Regression	16.96	4	4.24	31.24	4.09E-07	2.06
Residual	0.99	15	0.066			0.257	Residual	2.04	15	0.136			0.368
**POS-C24-P24**	**SS**	**DF**	**MS**	**F**	**p**	**SD**	**NEG-C24-P24**	**SS**	**DF**	**MS**	**F**	**p**	**SD**
Total corr.	19	19	1			1	Total corr.	19	19	1			1
Regression	18.17	4	4.54	82.33	5.08E-10	2.13	Regression	17.85	6	2.97	33.61	3.48E-07	1.72
Residual	0.83	15	0.055			0.235	Residual	1.15	13	0.089			0.298
**POS-P24-M24**	**SS**	**DF**	**MS**	**F**	**p**	**SD**	**NEG-P24-M24**	**SS**	**DF**	**MS**	**F**	**p**	**SD**
Total corr.	19	19	1			1	Total corr.	19	19	1			1
Regression	14.62	6	2.44	7.24	1.46E-03	1.56	Regression	10.31	10	1.03	1.07	4.65E-02	1.02
Residual	4.38	13	0.337			0.580	Residual	8.69	9	0.965			0.982

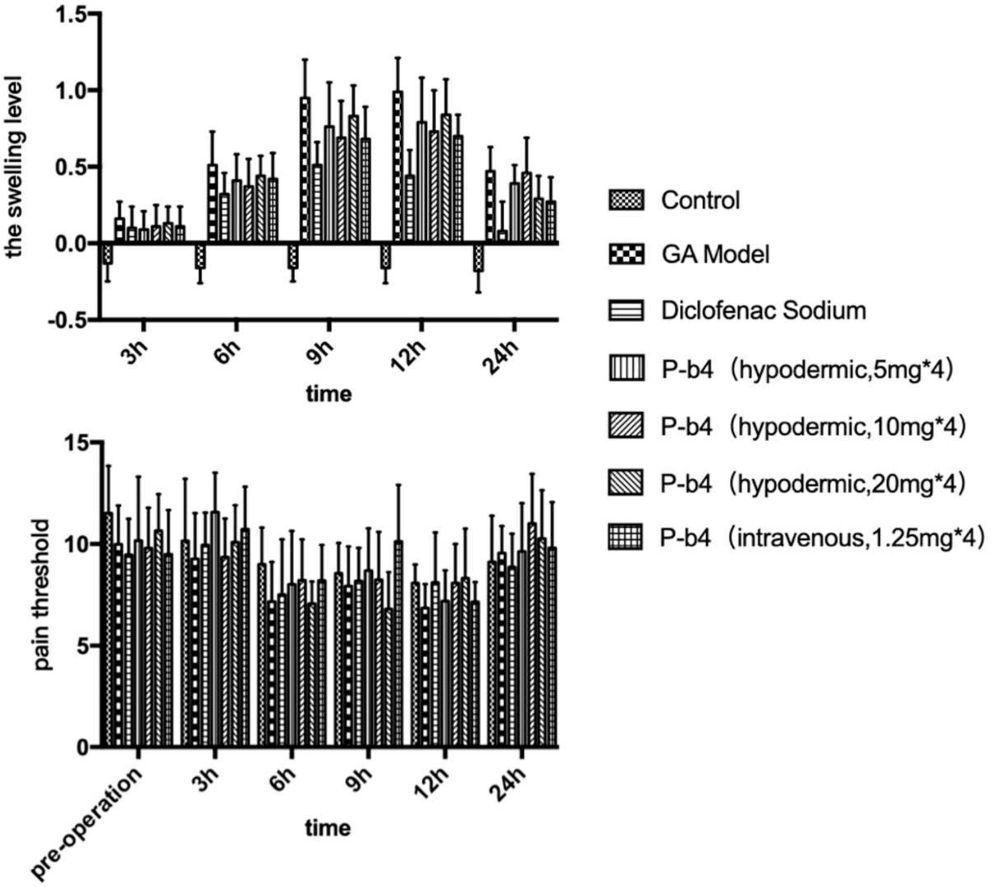

Figure S1. The bar plot of the value of each group on toes/ankle swelling and pain threshold.

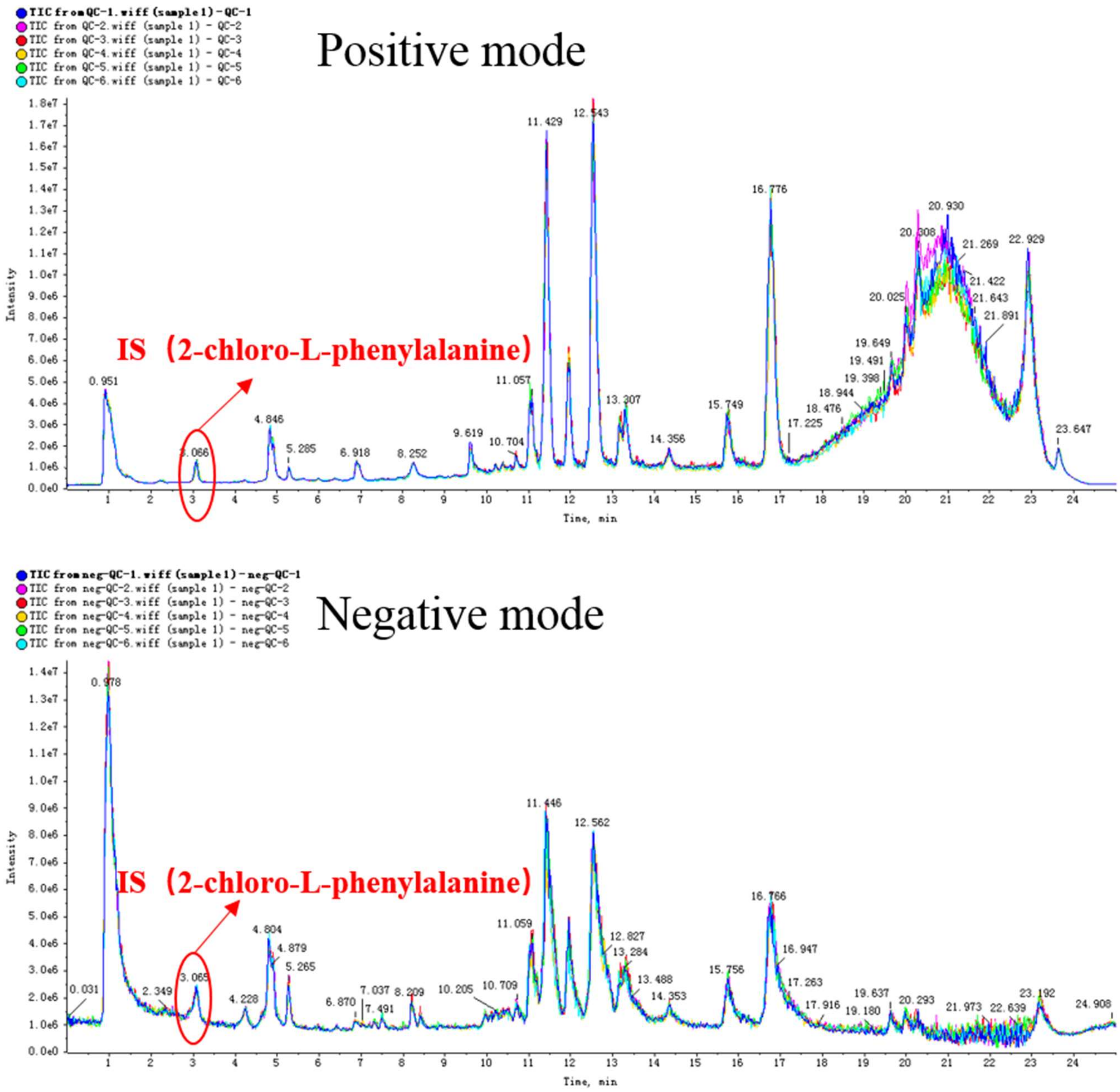

Figure S2. TICs of 6 QC samples.

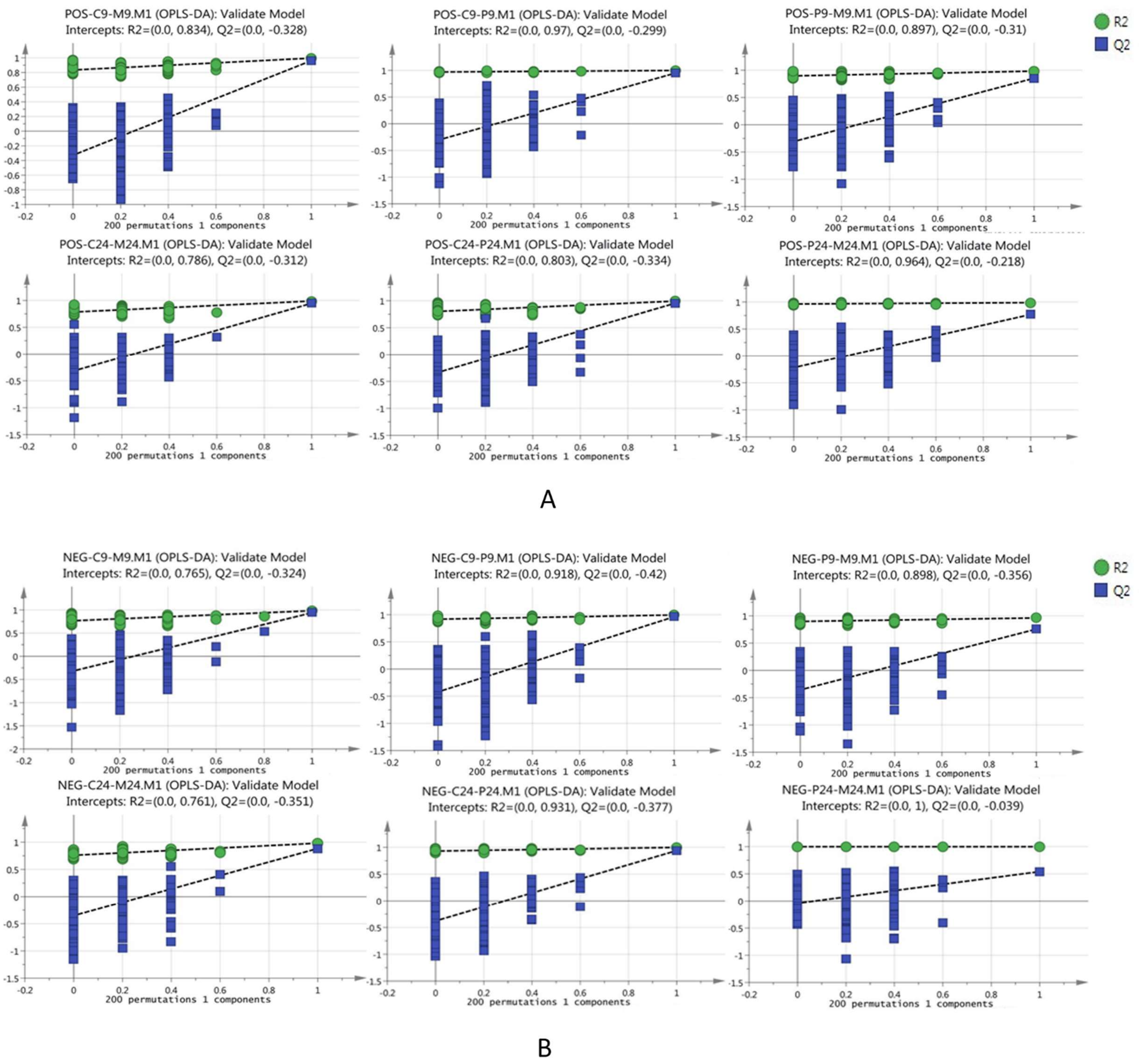

Figure S3. The permutation of 200 tests of 12 OPLS-DA models.A: positive ion mode;B: negative ion mode.
